# Usual source of care and access to care in the US: 2005 vs. 2015

**DOI:** 10.1371/journal.pone.0278015

**Published:** 2023-01-13

**Authors:** De-Chih Lee, Leiyu Shi, Jing Wang, Gang Sun

**Affiliations:** 1 Department of Information Management, Da-Yeh University, Changhua, Taiwan, R.O.C; 2 Johns Hopkins Primary Care Policy Center, Johns Hopkins Bloomberg School of Public Health, Baltimore, MD, United States of America; 3 Johns Hopkins Bloomberg School of Public Health, Baltimore, Maryland, United States of America; 4 Anhui Medical University, Hefei, China; 5 Department of Health Management, School of Health Management, Southern Medical University, Guangzhou, Guangdong, China; Osakidetza Basque Health Service, SPAIN

## Abstract

**Introduction:**

The study examined the association of usual source of care (USC) and healthcare access using a series of access indicators including both positive and negative measures for the US population in 2005 and 2015 while controlling for individual sociodemographic and socioeconomic characteristics. Results of the study would help advance the knowledge of the relationship between USC and access to care and assist decisionmakers in targeted interventions to enhance USC as a strategy to enhance access.

**Methods:**

The household component of the US Medical Expenditure Panel Survey (MEPS-HC) in 2005 and 2015 were used for the study. To estimate the relative risk of having USC on access to care, odds ratios (ORs) and their 95% confidence intervals (CIs) were used with unconditional logistic regression and adjusted for socioeconomic and demographic characteristics.

**Results:**

Those with USC were significantly more likely to have better access to care compared to those without USC. The USC-access connection remains significant and strong even after controlling for socioeconomic and demographic characteristics. Regarding subpopulations likely to lack USC, two notable findings are that racial/ethnic minorities (Black, Asian, and Hispanic) are more likely than White to lack USC and that those uninsured are more likely to lack USC.

**Conclusion:**

The study contributes to the literature on USC and access to care and has significant policy and practical implications. For example, having a USC is critical to accessing the health system and is particularly important as a tool to addressing racial disparities in access.

## Introduction

If there is a place or provider an individual or family usually goes when sick or in need of advice about health, that place or healthcare provider is referred to as usual source of care (USC) [1]. In the US, USC can be a provider such as primary care physician or a facility such as a physician group practice [2].

USC is important in many ways as it is usually related to enhancing healthcare access, improving quality of care services, lowering healthcare expenditures, and decreasing healthcare disparities [3]. The “Access to Health Services” topic of Healthy People 2020 mentions that ensuring individuals to have a usual and ongoing source of care can help improve people’s access to healthcare services [4]. Having a primary care provider (PCP) who serves as USC is especially crucial in increasing service access and improving service quality because PCPs directly provide integrated services to patients and develop sustainable relationship with patients [4].

Over the years, many studies have demonstrated that having a USC is positively correlated with individual’s access to healthcare services. An earlier study conducted by the Institute of Medicine in late 1990s points out that people with USC usually have better access to primary care than those who do not [5]. Other studies show that individuals are more likely to use clinical prevention services such as cancer screening and immunization if they have USC, which helps healthcare providers know patients’ needs more comprehensively and provide patient-centered care accordingly [6,7]. A cross-sectional study among 48,720 adults from the Community Tracking Household Survey assesses access to healthcare and quality of care by having or not having a USC; it shows that people with continuous USC are less likely to have access issues in terms of unmet medical needs and postponed medical care [6]. In addition to providing timely access to healthcare, USC also acts as a coordinator to help individuals gain access to more specialized healthcare providers [6]. A more recent study by Levine et al. shows that patients with primary care as USC received better outpatient care than those without primary care (25).

The number of people having USC in the US has been floating up and down over the past decade. In 2001, 18.4% US population reported that they did not have USC when sick. In 2017, 20.2% of population reported not having USC [8,9]. Overall, this indicates that there are fewer people having USC nowadays despite the improvement of healthcare access following the pass of Affordable Care Act in 2010 and increase in health insurance coverage. The estimated percentage of the US civilian noninstitutionalized population having USC decreased from 80% to 76% from 2015 to 2016 [10–12]. A recent study by Liaw et al. focuses on the trend of USC types and examines the personal characteristics associated with different USC types (26). The authors noted that a growing number of respondents were reporting facilities as their USC or none at all. Those with No USC and Facility USC increased 10% and 18%, respectively, while those with Person USCs decreased by 43% (26).

Although studies have demonstrated the significant association between USC and healthcare access, there were a number of limitations. First, indicators of access were limited focusing mainly on one practice setting such as physician office or hospital outpatient rather than addressing all types of settings where patients may receive care. Other settings of care such as community health centers or even ER are often not studied even though they represent USC for many with Medicaid or uninsured. Access to prescription drugs including refills is critical for patients particularly those with chronical health conditions but was seldom studied. There was also a lack of study between USC and *lack* of access such as unable to get necessary medical, dental, or prescription drug services. Second, many studies have not controlled for other correlates of access including individual predisposing, enabling, and need characteristics. These sociodemographic and socioeconomic factors could moderate or exacerbate the impact of USC on access and should be taken into account. Third, most prior research has not examined the *consistent* relationship between USC and access typically focusing on point-of-time analysis rather than examining the relationship for the same population across different time periods. Repeated analysis for the same population over time could strengthen the conclusion if the findings are consistent over time.

This study overcomes these limitations by examining the association of USC and healthcare access using a series of access indicators including both positive and negative measures for the US population in 2005 and 2015 while controlling for individual sociodemographic characteristics. Results of the study would help advance the knowledge of the relationship between USC and access to care which could assist decisionmakers such as policymakers and insurance companies to prioritize resources and design health plans accordingly.

## Methods

### Data

The household component of the US Medical Expenditure Panel Survey (MEPS-HC) in 2005 and 2015 were used for the study. Specifically, analyses were limited to the nonelderly adult respondents (those age 18–64). Children (< 18) and elderly (65+) were excluded due to the nature of the survey which was limited to adult residents for certain access related questions and the fact that the elderly were covered by Medicare which typically mandates USC. The MEPS-HC is a nationally representative survey of the US civilian non-institutionalized population which provides nationally representative estimates of healthcare use, expenditures, payment sources, and health insurance coverage. Data of two years (2005 and 2015) were downloaded from the AHRQ website (https://meps.ahrq.gov/survey_comp/household.jsp.). The year 2015 was chosen because it was the latest year complete access to care measures were collected and available at the time of our analyses and the access to care measures were similarly defined to earlier years. The year 2005 was chosen to allow us to track progress over a ten-year span. Since MEPS datasets are released periodically, future analyses may corroborate our findings when more current years of data were released.

### Measures

#### Usual source of care

Each adult respondent was asked whether there was a particular doctor’s office, clinic, health center, or other place that he/she usually went to if he/she was sick or needed advice about his/her health. Based on the MEPS definition, USC referred to the particular medical professional, doctor’s office, clinic, health center, or other place where a person would usually go if sick or in need of advice about his or her health (https://meps.ahrq.gov/mepsweb/data_stats/MEPS_topics.jsp?topicid=44Z-1). Having no usual source of care was coded as “0” and having it coded as “1”.

Additionally, the main reason that a person didn’t have a USC provider was analyzed which included seldom or never get sick, recently moved to area, don’t know where to go, USC in area not available, can’t find provider who speaks the same language, go to different places for different needs, just changed insurance plans, don’t use doctors/treat self, cost of medical care, no health insurance, and other reason.

#### Access to care

Access to care was measured partly by utilization of medical services including office-based provider visits, outpatient department visits, emergency-room visits, hospital discharges, and prescription medications including refill. Visits to office-based providers consisted of encounters that took place primarily in office-based settings and clinics whether a physician or non-physician provider was seen. Visits to hospital outpatient departments included visits whether a physician or non-physician provider was seen. A count of all emergency room visits reported for the survey year was used. Since emergency room is not a place that would be considered an effective or appropriate access point for primary care, preventive services, or anything other than acute care or emergencies, it was included as a measure of poor or inappropriate access. The total number of nights was associated with the total discharges of inpatients. A count of all prescribed medications purchased during the survey year was collected through the household questionnaire and a pharmacy component survey. For logistic regression, zero visit was recoded as “0” and 1 or more visits recoded as “1.”

In addition to the actual counts of utilization, access to care was also measured by respondents’ self-perceived barriers to receiving care, namely, medical, dental, and prescription medicine services. We coded as “0” when the respondent needed service but was unable to receive it and “1” to indicate either the respondent did not need service or needed service and was able to receive it. Kirby JB et al. [13] found that 9.91% and 10.24% of population under age 65 in the US metro and non-metro counties had unmet need in 2014–2015, in which individuals who reported having unmet need or delays with respect to medical care, dental care, or obtaining prescription drugs were coded as having unmet need.

### Sociodemographics

Respondents’ demographic and socioeconomic characteristics were included as controls in multivariate analyses. These included sex (male or female); age (18–24, 25–44, or 45–64); marital status (married, never married, or widowed/divorced/separated); region (northeast, midwest, south, or west); education in years (0, 1–8, 9–11, 12, or 13+); race/ethnicity (White only, Black only, American Indian/Alaska Native, Asian only, Hispanic, or Others); poverty status (‘poor’ corresponding to <100% FPL, ‘near poor’ corresponding to 100%-124% FPL, ‘low income’ corresponding to 125%-199% FPL, ‘middle income’ corresponding to 200%-399% FPL, ‘high income’ corresponding to 400%+ FPL); and health insurance (private insurance, Medicare, Medicaid, other insured, or uninsured).

### Analysis

Data were analyzed using SPSS 23.0 software (IBM Corp, Armonk, NY, USA). For comparative analysis between those with USC and without, proportions were calculated among different sub-categories of access and different sociodemographic subpopulations. The Chi-squared test was applied to compare the differences. To estimate the relative risk of having USC on access to care, odds ratios (ORs) and their 95% confidence intervals (CIs) were used with unconditional logistic regression and adjusted for socioeconomic and demographic characteristics. Univariate and multivariate analyses were performed weighted by the variable “PERWT.” P-value no more than 0.05 was regarded as significant difference.

## Results

### Descriptive analysis

For the years studied (2005 and 2015), the proportion of American adults under age 65 without USC remained about the same at 21.7% and 21.2%, respectively. The top reasons for not having USC included cost of medical care, recently moved to area, go to different places for different needs, don’t know where to go, USC not available, and health insurance related.

In terms of access to care measures, the mean of office-based provider visits increased slightly from 4.47 in 2005 to 4.83 in 2015; while mean of hospital discharges decreased from 0.57 in 2005 to 0.44 in 2015 (Table 1). There was little change with respect to outpatient visits, emergency room visits, and prescription medications refills between 2005 and 2015. The proportions of respondents with no outpatient visits, hospitalization, and prescription refills increased from 2005 to 2015 (86.1% to 86.7%, 92.6% to 94.0%, and 41.8% to 44.7%, respectively). The proportions of respondents unable to get necessary medical, dental, and prescriptions decreased somewhat between 2005 and 2015 (2.8% to 1.9%, 4.7% to 3.7%, and 2.1% to 1.6%, respectively).

**Table 1:** Measures of access to care: 2005 vs. 2015.

Measures of Access to Care	2005 Mean ± SD, n (%)	2015 Mean ± SD, n (%)
Office-based provider visits	4.47 ± 9.68, 11124 (32.8%)	4.83 ± 11.16, 11405 (32.2%)
Outpatient dept provider visits	0.42 ± 2.82, 29248 (86.1%)	0.40 ± 2.52, 30709 (86.7%)
Emergency room visits	0.19 ± 0.57, 29205 (86.0%)	0.20 ± 0.62, 30392 (85.8%)
Hospital discharges	0.57 ± 4.39, 31458 (92.6%)	0.44 ± 4.24, 33313 (94.0%)
Prescription medications including refills	9.35 ± 19.50, 14187 (41.8%)	9.33 ± 20.30, 15823 (44.7%)
	2005 n (%)	2015 n (%)
Unable to get necessary medical care	967 (2.8%)	689 (1.9%)
Unable to get necessary dental care	1600 (4.7%)	1300 (3.7%)
Unable to get necessary prescription medications	708 (2.1%)	573 (1.6%)

### Comparative analysis

Table 2 compares respondents’ sociodemographic characteristics between those with USC and without for 2005 and 2015, respectively. For both years, measures of sex, age, marital status, region, education, race, poverty status, and insurance were significantly related to the proportion of having USC or not (p<0.001). In general, females were more likely to have USC than males (84.3%-83.9% vs. 76.2%-76.5%), older adults (age 45–64) more than younger adults (18–24) (83.9%-82.6% vs. 62.5%-68.4%), married more than widowed, divorced, or separated (81.1%-80.9% vs. 67.4%-67.4%), Northeast residents more than South residents (87.2%-85% vs. 77.4%-76.7%), college-educated (13+ years) more than high-school educated (12 years) (79.4%-77.8% vs. 77.4%-73.3%), White (84.2%-83%) more than Black (76.3%-77.3%), Asian (72.5%-77.7%) or Hispanic (67.6%-73.2%), and higher-income (400%+ FPL) than lower-income (<100% FPL) (85.4%-83.8% vs. 74.3%-76.4%).

**Table 2:** Usual source of care by sociodemographic characteristics: 2005 vs. 2015.

Socio- Demographic Characteristics	2005		2015	
	Having USC (%)	Having no USC (%)	Having USC (%)	Having no USC (%)
SEX				
Male	108198682 (76.2%)	33724301 (23.8%)*	116795028 (76.5%)	35908645 (23.5%)*
Female	124539710 (84.3%)	23258297 (15.7%)	133726007 (83.9%)	25640894 (16.1%)
AGE				
18–24	17627297 (62.5%)	10559227 (37.5%)*	19699102 (68.4%)	9104612 (31.6%)
25–44	55572824 (68.7%)	25281307 (31.3%)	52085604 (63.8%)	29560105 (36.2%)*
45–64	61392502 (83.9%)	11811346 (16.1%)	68158676 (82.6%)	14337030 (17.4%)
MARITAL STATUS				
Married	98625944 (81.1%)	22980899 (18.9%)	103438352 (80.9%)	24454607 (19.1%)
Never married	35081693 (81.0%)	8255707 (19.0%)	36496079 (82.4%)	7786654 (17.6%)
Widowed, divorced, separated	42018269 (67.4%)	20358794 (32.6%)*	52140821 (67.4%)	25202141 (32.6%)*
REGION				
Northeast	46534852 (87.2%)	6804229 (12.8%)	46503193 (85.0%)	8185788 (15.0%)
Midwest	53383962 (82.9%)	11005608 (17.1%)	55742012 (84.1%)	10530984 (15.9%)
South	80653076 (77.4%)	23512325 (22.6%)	89923782 (76.7%)	27251312 (23.3%)*
West	52031881 (77.2%)	15408975 (22.8%)*	58225717 (79.0%)	15450639 (21.0%)
EDUCATION				
0 year	9054220 (90.2%)	984621 (9.8%)	4526590 (91.7%)	408328 (8.3%)
1–8 years	38081784 (83.3%)	7636305 (16.7%)	20615488 (87.0%)	3092760 (13.0%)
9–11 years	25157845 (73.6%)	9006204 (26.4%)*	13087739 (78.5%)	3579098 (21.5%)
12 years	53371896 (77.4%)	15574973 (22.6%)	28809722 (73.3%)	10510514 (26.7%)*
13+ years	84218975 (79.4%)	21884906 (20.6%)	55616977 (77.8%)	15839151 (22.2%)
RACE				
White only	162272227 (84.2%)	30352192 (15.8%)	156638344 (83.0%)	32139775 (17.0%)
Black only	26567437 (76.3%)	8270683 (23.7%)	29442902 (77.3%)	8669774 (22.7%)
American Indian/Alaska Native	1469630 (81.0%)	343879 (19.0%)	2315192 (79.4%)	598998 (20.6%)
Asian only	8925176 (72.5%)	3392028 (27.5%)	13194521 (77.7%)	3792733 (22.3%)
Hispanic	28720528 (67.6%)	13743341 (32.4%)*	39969686 (73.2%)	14610800 (26.8%)*
Others	4783393 (84.5%)	880456 (15.5%)	8960389 (83.8%)	1737460 (16.2%)
POVERTY STATUS				
<100% FPL	27316590 (74.3%)	9435049 (25.7%)*	32118969 (76.4%)	9948223 (23.6%)*
100%-124% FPL	9700736 (75.9%)	3082878 (24.1%)	10560846 (77.7%)	3030341 (22.3%)
125%-199% FPL	30804811 (77.0%)	9226166 (23.0%)	32553642 (76.3%)	10115615 (23.7%)
200%-399% FPL	71539269 (78.8%)	19232693 (21.2%)	70996813 (79.5%)	18258780 (20.5%)
400%+ FPL	93376985 (85.4%)	16005813 (14.6%)	104290765 (83.8%)	20196582 (16.2%)
INSURANCE				
Private	50234861 (92.2%)	4273828 (7.8%)	81666025 (90.7%)	8330429 (9.3%)
Medicaid	2991577 (95.5%)	139513 (4.5%)	16536567 (88.9%)	2057222 (11.1%)
Other insured	7390831 (90.4%)	783273 (9.6%)	6055495 (94.4%)	356988 (5.6%)
Uninsured	15898137 (46.8%)	18104929 (53.2%)*	1550082 (81.3%)	355765 (18.7%)*

*: P<0.001 based on Chi-square tests among subcategories of a socio-demographic characteristic in the same year.

Table 3 compares access to care performance between those with USC and without for 2005 and 2015, respectively. For both years, compared to those without USC, those with USC were significantly more likely to report office-based visits, outpatient visits, ER visits, hospitalization, and prescription drug refills. Comparing the two years, we found that the proportions of those with no outpatient visits, no ER visits, and no prescription medication including refill increased from 80.3%, 91.9%, and 27.1% in 2005 to 81.4%, 93.6%, and 29.1% in 2015, respectively, among those with USC.

**Table 3:** Association between Usual Source of Care (USC) and access to care: 2005 vs. 2015.

Access to Care Measures	2005		2015	
	With USC	Without USC	With USC	Without USC
Office-based provider visits				
0 (%)	28502273 (21.2%)	27352128 (57.4%)*	27825445 (19.9%)	28868806 (54.5%)*
1+ (%)	106090350 (78.8%)	20299752 (42.6%)	112117836 (80.1%)	24132941 (45.5%)
Outpatient dept provider visits				
0 (%)	108087325 (80.3%)	44804436 (94.0%)*	113874149 (81.4%)	49396915 (93.2%)*
1+ (%)	26505298 (19.7%)	2847444 (6.0%)	26069132 (18.6%)	3604832 (6.8%)
Emergency room visits				
0 (%)	115979634 (86.2%)	42546853 (89.3%)*	119872578 (85.7%)	47277811 (89.2%)*
1+ (%)	18612989 (13.8%)	5105027 (10.7%)	20070703 (14.3%)	5723936 (10.8%)
Hospital discharges				
0 (%)	123728097 (91.9%)	45471535 (95.4%)*	130968211 (93.6%)	51234482 (96.7%)*
1+ (%)	10864526 (8.1%)	2180345 (4.6%)	8975071 (6.4%)	1767265 (3.3%)
Prescription medications including refills				
0 (%)	36490148 (27.1%)	30215249 (63.4%)**	40731146 (29.1%)	33612022 (63.4%)**
1+ (%)	98102475 (72.9%)	17436631 (36.6%)	99212136 (70.9%)	19389725 (36.6%)
Unable to get necessary medical care				
Yes (%)	4024444 (3.0%)	2660115 (5.6%)*	3672928 (2.6%)	1317840 (2.5%)
No (%)	130494483 (97.0%)	44854086 (94.4%)	136094736 (97.4%)	51572170 (97.5%)
Unable to get necessary dental care				
Yes (%)	6619949 (4.9%)	3530927 (7.4%)*	6219473 (4.5%)	2805185 (5.3%)*
No (%)	127824130 (95.1%)	43962485 (92.6%)	133364078 (95.5%)	50051156 (94.7%)
Unable to get necessary prescription medications				
Yes (%)	3264254 (2.4%)	1501075 (3.2%)*	3064231 (2.2%)	777975 (1.5%)
No (%)	131209696 (97.6%)	46024587 (96.8%)	136705407 (97.8%)	52125930 (98.5%)

*: P<0.001 based on Chi-square test between those with USC and those without on each access to care measure.

In terms of the negative measures of access to care, compared to those without USC, those with USC were significantly less likely to feel unable to get necessary medical care (3% vs. 5.6% in 2005), dental care (4.9% vs. 7.4% in 2005 and 4.5% vs. 5.3% in 2015), and prescription medications (2.4% vs. 3.2% in 2005). Compared to 2005, those felt unable to get necessary medical, dental, or prescription services declined slightly indicating some progress made removing access barriers between 2005 and 2015.

### Regression analysis

Logistic regressions were performed to examine the association of USC with access to care measures as dependent variables. Odds ratios and their 95% confidence intervals were provided for both the unadjusted and adjusted models (see Table 4). The results showed that those with USC were significantly more likely to have better access to care compared to those without USC for both 2005 and 2015. This is the case even after controlling for socioeconomic and demographic characteristics. Compared to those without USC, those with USC were 3.3 times more likely to have office-based provider visits in 2005 (3.5 times in 2015), 2.4 times more likely to have hospital outpatient visits in 2005 (1.2 times in 2015), 1.2 times more likely to hospitalization in 2005 (1.3 times in 2015), 3.9 times more likely to have prescription medications including refills in 2005 (2.6 times in 2015), 1.3 times less likely to feel unable to get medical care in 2005 (1.4 times in 2015), 1.2 times less likely to feel unable to get dental care in 2005 (1.04 times in 2015), and 1.3 times less likely to feel unable to get prescription medications in 2005 (1.2 times in 2015). The only measure that bent this pattern was ER visits where those with USC were 1.5 times more likely to have ER visits than those without USC in 2005 but 3% less likely in 2015.

**Table 4:** Logistic regression on Usual Source of Care (USC) by access to care: 2005 vs. 2015.

Dependent Variables: *access to care measures (0 vs*. *1)*	Independent Variable: USC (1 vs.0)			
	2005		2015	
	Unadjusted OR (95% CI)	Adjusted# OR (95% CI)	Unadjusted OR (95% CI)	Adjusted# OR OR (95% CI)
Office-based provider visits (0 = no visit, 1 = 1+ visit)	5.015* (5.012–5.019)	3.314* (3.309–3.319)	4.820* (4.817–4.823)	3.513* (3.505–3.522)
Outpatient dept provider visits (0 = no visit, 1 = 1+ visit)	3.859* (3.854–3.863)	2.363* (2.357–2.368)	3.137* (3.133–3.141)	1.168* (1.165–1.171)
Emergency room visits (0 = no visit, 1 = 1+ visit)	1.338* (1.336–1.339)	1.527* (1.524–1.530)	1.383* (1.382–1.384)	0.973* (0.971–0.975)
Hospital discharges (0 = no discharge, 1 = 1+discharge)	1.831* (1.829–1.834)	1.163* (1.160–1.166)	1.987* (1.983–1.990)	1.304* (1.299–1.308)
Prescription medications including refills (0 = no medication, 1 = 1+ medication)	4.659* (4.655–4.662)	3.948* (3.942–3.955)	4.222* (4.220–4.225)	2.572* (2.565–2.580)
Unable to get necessary medical care (0 = unable, 1 = able)	1.923* (1.920–1.926)	1.290* (1.287–1.293)	0.947* (0.945–0.949)	1.381* (1.375–1.387)
Unable to get necessary dental care (0 = unable, 1 = able)	1.551* (1.549–1.553)	1.146* (1.144–1.148)	1.202* (1.200–1.204)	1.036* (1.032–1.040)
Unable to get necessary prescription medications (0 = unable, 1 = able)	1.311* (1.308–1.314)	1.280* (1.276–1.283)	0.666* (0.664–0.668)	1.207* (1.200–1.213)

*: P<0.001. #: Adjusted for personal socio-demographic characteristics such as sex, age, marital status, region, education, race/ethnicity, poverty status, and insurance.

## Discussion

Although much research has been conducted regarding USC and its influence on access to care, ours is among the few that uses a representative US population, employs both positive and negative measures of access, and controls for sociodemographic and socioeconomic characteristics while assessing the association between USC and access to care. We found that the proportions of US adults aged of 18–64 having no USC was 21.7% and 21.2% in 2005 and 2015, respectively. This persistent pattern of lacking USC indicates the failure of recent healthcare reforms that expand insurance coverage but not USC. Although no conclusive evidence in the dataset allowed us to identify reasons for lack of USC, other studies seem to suggest that “cost of medical care” [14] and ‘lack of health insurance’ [15–20] could be the primary factors. Hong et al., based on US adults aged 18 years or older who had health insurance coverage for a full year, found that the effect of USC on perceived access to care and addressing unmet needs was significantly enhanced [21].

The results of the analyses showed that those with USC were significantly more likely to have better access to care compared to those without USC. Our results further confirmed the bulk of research linking USC with enhanced access [21–23]. Moreover, our study noted that the USC-access connection remains significant and strong even after controlling for socioeconomic and demographic characteristics. These results (especially the high odds ratios between those with and without USC with regard to access indicators) are consistent for both 2005 and 2015 analyses indicating the strong association between USC and access to care. The results also showed that access to care remained generally the same between 2005 and 2015 with some measures deteriorating and others improving. Comparing the two years, the proportions of those with no outpatient visits, no ER visits, and no prescription medications including refills increased from 80.3%, 91.9%, and 27.1% in 2005 to 81.4%, 93.6%, and 29.1% in 2015, respectively, among those with USC. On the other hand, those felt unable to get necessary medical, dental, or prescription services declined slightly indicating some progress made removing access barriers between 2005 and 2015.

The influence of USC on primary care visits further enhanced between 2005 and 2015. Those with USC were 3.5 times more likely to have office-based provider visits (primarily provided by primary care physicians) in 2015 (compared to 3.3 times in 2005) and 1.4 times less likely to feel unable to get medical care in 2015 (1.3 times in 2005). On the other hand, those with USC were 3% less likely to have ER visits in 2015 (1.5 times more likely in 2005) showing the inverse relationship between USC and ER visits. This is consistent with prior study that showed USC increases visits to primary care doctors but decreases visits to ER [24] since ER visits were generally not considered an effective or appropriate access point for primary care, preventive services, or anything other than acute care or emergencies.

Another important contribution of our study is to identify subpopulations likely to lack USC. Two notable findings are that racial/ethnic minorities (Black, Asian, and Hispanic) are more likely than White to lack USC and that those uninsured are more likely to lack USC. Moreover, the magnitudes of the differences (7–8% for White vs. Black, 6–12% for White vs. Asian, and 10–17% for White vs. Hispanic and 13–44% for insured vs. uninsured) suggests that race/ethnicity and insurance coverage are key areas for policy interventions in order to expand access.

These findings have significant policy and practical implications. For example, having a USC is critical to accessing the health system and addressing racial and socioeconomic disparities in access to care. Compared to other industrialized countries, the US underperforms on key indicators of primary care [25] including number of primary care physicians per capita, rates of primary care visits, and in the share of Americans having USC. Policymakers can enhance investment towards primary care by increasing the availability and supply of primary care, especially for low-income communities, through programs like the National Health Service Corps, elevating the payment scale, and enabling providers to deliver ongoing, comprehensive, and high-quality care through sustained patient-provider partnership in a USC model. A strong relationship with the usual source of care matters now more than ever to save lives in an increasingly daunting health crisis.

There are a number of limitations of the study. First, the cross-sectional nature of the datasets does not allow causal analysis. While we demonstrate that USC is associated with positive access to care, we cannot assume that the relationship is causal and therefore interpretation must be made cautiously. Second, while we perform racial disparity analyses, the limited sample size does not allow up to study various subpopulations within the large racial designation. For example, Asians include people from two dozens of Asian countries with diverse background and culture. People with multiracial background, a growing phenomenon in the US, cannot be distinguished from the dataset. Finally, due to inconsistency in measurement over time and missing values for key measures, our study only uses limited number of years in the analysis. However, despite these limitations, our study makes a contribution to the field by asserting the positive association between different types of USC and various access to care measures for people with different racial and socioeconomic background.
